# Design and analysis of a dual-port multiband RF-to-DC converter for IoT energy harvesting applications

**DOI:** 10.1371/journal.pone.0321729

**Published:** 2025-05-13

**Authors:** Bismah Tariq, Muhammad Amjad, Khaled A. Al-Jaloud, Waqar Ahmad Malik, Syed Tamoor Shah, Rifaqat Hussain

**Affiliations:** 1 Faculty of Engineering & Technology, The Islamia University of Bahawalpur, Pakistan; 2 College of Engineering, Muzahimiyah Branch, King Saud University, P.O. Box 2454, Riyadh 11451, Saudi Arabia; 3 Department of Avionics Engineering, CAE, NUST Risalpur, Pakistan; 4 SEECS, National University of Science & Technology (NUST), Pakistan; 5 School of Electronic Engineering and Computer Science, Queen Mary University of London, United Kingdom; Model Institute of Engineering and Technology, INDIA

## Abstract

Internet of Things (IoT) demands efficient and sustainable energy sources for autonomous power systems. This article presents the design and analysis of a Dual-Port Multiband RF-to-DC Converter employing a multi-stage Cockcroft Walton Voltage Multiplier (CWVM) topology, which is optimized for IoT energy harvesting applications. By utilizing L-network and π-network impedance matching with distributed elements, the converter effectively harvests RF energy across six frequency bands ranging from 0.87 to 2.5 GHz. The dual-port architecture enhances power output and provides redundancy, improving the reliability of the energy harvesting system. While, the multiband feature improves the versatility of energy acquisition. The converter achieves a peak efficiency of 66% and 62% at 10 kΩ and 18 kΩ, respectively. The performance of the proposed design is comprehensively analyzed and optimized, considering the impedance matching, output voltage, and conversion efficiency. The performance of the proposed design is compared with recent converter designs in the literature, which shows that this research is a valuable contribution to the ongoing development of energy-efficient solutions. This work has significant implications for powering low-power sensors, wireless sensor networks, and other IoT devices in diverse environments, addressing the need for prolonged autonomy and reduced reliance on traditional power sources.

## 1 Introduction

The Internet of Things (IoT) is a transformative technology where everyday objects are inculcated with digital intelligence, enabling them to connect, communicate, and interact with each other and with humans. This interconnected web of devices and sensors, from smart thermostats to industrial machinery, has revolutionized various industries, enhancing efficiency, convenience, and data-driven decision-making [1].

However, the rapid propagation of IoT devices is accompanied by a significant challenge of surge in energy demands [2]. To overcome this challenge, Wireless power transfer (WPT) and RF energy harvesting (RFEH) [3, 4], are becoming vital focus for more research now a days. These are the favorable technologies to power up wireless IoT devices [5] and ensure their continuous operation and connectivity. RFEH harnesses ambient radio frequency signals to generate electrical power, enabling sustainable and autonomous operation of IoT devices. It is useful for wireless powering of remote, hard to access, low power devices such as health care and biomedical systems [6–8], wireless sensor networks [9, 10], IoT applications [11, 12], and wireless communications [13].

The sources of ambient RF energy are TV/radio broadcast stations, mobile base stations, handheld stations, WiFi and Bluetooth devices, etc. [14]. As the ambient RF energy is present at different frequency bands, multiband RF-to-DC converters are desirable to combine multiple frequency bands for energy harvesting, enhancing versatility and efficiency in powering IoT devices, while reducing reliance on single-band sources and improving overall energy harvest capabilities [15].

Some single band converters are reported in the literature for energy harvesting applications [16–18]. A single band converter working at frequency band of GSM-900 with efficiency 29.85% using HSMS285C Cockcroft-Walton voltage multiplier (VM) was proposed in [16]. Another converter operating at 2.5 GHz band was proposed which has provided efficiency of 66% using HSMS2852 single stage VM [17]. A converter operating at 5G frequency band of 3.5GHz band exhibited efficiency of 42.5% using HSMS286C Greinacher VM and radial stub matching [18].

Likewise dual band converters are reported in [19–21]. In [19], the proposed dual-band converter operates on 2.1 and 2.45 GHz bands with the power conversion efficiencies of 24% and 18% respectively. The matching was achieved using multiple stubs. In [20], the proposed dual-band operates on GSM-1800 and WiFi 2.4 bands, with the conversion efficiencies of 41% and 20%, respectively. A dual-band converter was presented in [21] for GSM bands with efficiencies upto 13% at –30 dBm. The converter was implemented by single series topology and pi-matching network.

Similarly, a triple-band converter was presented in [22] with efficiency upto 42%. While a converter proposed in [23] operates on GSM-900, GSM-1800 and UMTS-2100 bands with efficiency upto 40% and output voltage of 600mV. Also some multiband converters like quad-band [24–26], six band [27] and seven band [28] are also recently reported with efficiencies ranging from 8% upto around 40% for different designs.

Some broad-band converters are also found in literature [29–31]. The converter proposed in [29] operates on 0.87–2.7 GHz band providing overall PCE of 30%. The design was implemented using voltage-doubler configuration. Two broad-band converters were proposed, operating for 1–2.4 GHz with efficiency up to 50% [30] and for 1.9–2.5 GHz with efficiency of 32% [31].

The power conversion efficiency (PCE) of an energy harvesting converter is a figure of merit for its performance evaluation [3]. Power conversion efficiency enhancement of the converter at low power levels and over a wide range of frequencies is the key challenge due to complexities involved in achievement of simultaneous impedance matching at multiple frequencies.

In this paper, a dual-port, hexa-band converter is proposed, operating on 870 MHz, 1770 MHz, 1850 MHz, 2.2 GHz, 2.41 GHz, and 2.5 GHz frequency bands. The impedance matching, output voltage and power conversion efficiency of the converter are optimized, simultaneously, on both parts of the converter. A novel configuration of multi stage Cockcroft Walton voltage multiplier (CWVM) topology, an L-matching network and a π-matching network are used to achieve required performance. It achieves power conversion efficiencies in the range of 23 to 66% in all the desired frequency bands.

Following sections include the design technique, configuration, results, performance, discussion and the concluding remarks for the proposed converter.

## 2 Design technique and configuration

**Fig 1:**
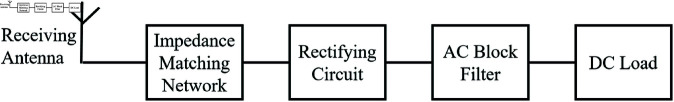
RF-to-DC converter block diagram.

The RF-to-DC converter consists of four basic working blocks as shown in Fig 1. The first block is an RF input block, which is the receiving wideband or a multiband antenna in practical scenarios. Next is the impedance matching block that consists of an impedance matching network (IMN) to ensure maximum power transfer and minimized signal reflection between the RF input block and the RF-to-DC rectification/ conversion block. The last block is the DC output block, which is usually a parallel combination of output load resistor and an AC block capacitor.

Many topologies are nowadays in practice for RF-to-DC rectification purposes as stated in the literature review. However, the voltage multiplier topologies are most commonly used in RF-to-DC converters for enhanced output DC voltage levels to compensate for the battery requirements of IoT devices.

Usually, the output from single stage voltage multiplier (VM) is extremely low because the received power (energy) is low, therefore, it is desirable to duplicate each stage of the VM circuit, to get a higher DC voltage from the input RF power signal. Fot this purpose, the multistage CWVM is advantageous as its output across each stage is equal to twice the peak input AC voltage ($V_{p}$) times the number of stages (N) being used, and the output can be obtained from any number of stages depending upon the application requirement [32].

**Fig 2:**
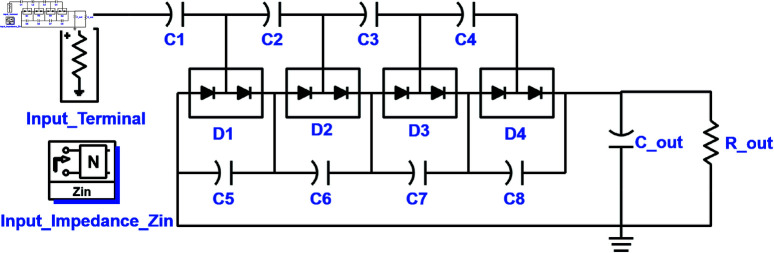
Schematic of 4-stage CWVM topology.

Hence the proposed scheme utilizes a 4-stage half-wave CWVM topology implemented by deploying 10 pF capacitors (C1-C8) and Schottky diode pair HSMS285C for frequency bands below 2 GHz and HSMS286C for frequency bands above 2 GHz, and shown in Fig 2. Cout is the output AC Block capacitor set as 27 pF. Schottky diode pairs are chosen because of their performance under low input power values, low voltage drop, fast switching, low saturation current, and junction capacitance. The dielectric substrate used for the design is a 0.78 mm thick sheet of Rogers 5880 with $\epsilon_{r}$ 2.2, tanσ 0.0009, and a copper thickness of 0.035 mm.

### 2.1 Single port converters design

To achieve the dual-port converter design, initially two single-port converters are designed using lumped components as well as distributed elements. Later these two single-port rectifying branches are integrated to yield the dual-port configuration and the IMNs are tuned for the hexa-band response. Following subsections discuss the single-port and dual-port designs in detail.

#### 2.1.1 Single port dual-band converter design

**Fig 3:**
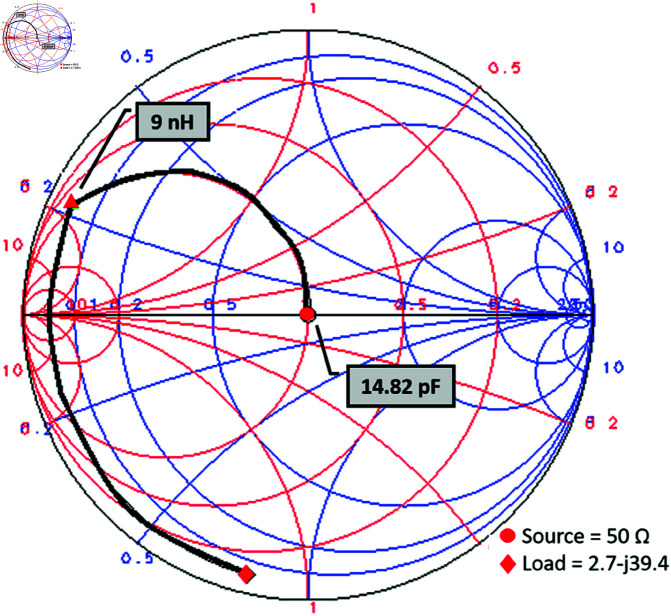
Impedance matching at 900 MHz using Smith chart.

The proposed single-port dual-band converter design is based on a single-port, single-band converter originally operating at 900 MHz. Later the modification of which yields the dual-band response for matching the converter impedance with that of the input source. An L-shape matching network, comprising the lumped components, is designed using Keysight ADS Smith chart utility. L-shape matching network is a single open stub network consisting of two reactive elements. One is the series inductive element, to provide impedance transformation and phase shift, and the other is shunt capacitive element, equivalent to the open stub. The input impedance (*Z*_*in*_) of the converter is noted against 900 MHz as 2.653–*j*39.864. The lumped element values are noted as *L* = 9*nH* and *C* = 14.82*pF* from the Smith chart as shown in Fig 3.

After designing the lumped element networks, these are converted to distributed elements, i.e. the microstrip and transmission lines. The lengths of the transmission line sections are found from Eq (1) [33]:

$$Z_{in}=Z_{o}\frac{Z_{L}+jZ_{o}tan\beta l}{Z_{o}+jZ_{L}tan\beta l}$$ (1)

where *Z*_*in*_, *Z*_*o*_, *Z*_*L*_, and β are the transmission line’s input impedance, the characteristic impedance, the load impedance, and the phase constant, respectively. The phase constant is calculated as Eq (2):

$$\beta =\frac{2\pi f}{v}$$ (2)

where *f* and *v* are the wave frequency and the speed of light in air. The input impedance *Z*_*inL*_ of an inductive element *L* and its corresponding transmission line section length *l*_1_ can be calculated by Eq (3)–(5);

$$Z_{inL}=jZ_{o}tan\beta l_{1}$$ (3)

$$j\omega L=jZ_{o}tan\beta l_{1}$$ (4)

$$L=\frac{Z_{o}tan\beta l_{1}}{\omega }$$ (5)

where ω is the angular frequency. On the other hand, the input impedance *Z*_*inC*_ of a capacitor, capable of capacitance *C*, and its corresponding transmission line’s length *l*_2_ is found by the Eq (6)–(8);

$$Z_{inC}=\frac{Z_{o}}{jtan\beta l_{2}}$$ (6)

$$\frac{1}{j\omega C}=\frac{Z_{o}}{jtan\beta l_{2}}$$ (7)

$$C=\frac{jtan\beta l_{2}}{\omega Z_{o}}$$ (8)

Remember that the $\beta l_{1}$ and $\beta l_{2}$ obtained from the above set of equations are the electrical lengths of the transmission line sections, which are further converted to physical lengths (mm) using the LineCalc tool in ADS. To calculate the width *W* of the transmission line sections, Eq (9)–(12) are used [34].

$$\frac{W}{h}=\frac{2}{\pi }\left\{\{\frac{\epsilon_{r}-1}{2\epsilon_{r}}[\ln\left(B-1\right)-\frac{0.61}{\epsilon_{r}}+0.39]+B-1-\ln\left(2B-1\right)\}\right\},\;for\;A>1.52$$ (9)

$$\frac{W}{h}=\frac{8e^{A}}{e^{2A}-2},\;forA<1.52$$ (10)

$$A=\frac{Z_{o}}{60}\sqrt{\frac{\epsilon_{r}+1}{2}}+\frac{\epsilon_{r}-1}{\epsilon_{r}+1}\left\{\frac{0.11}{\epsilon_{r}}+0.23\right\}$$ (11)

$$B=\frac{529.18}{Z_{o}\sqrt{\epsilon_{r}}}$$ (12)

Where *h* is the substrate height and both *A* and *B* are variables. In our case, we found *A* = 1.159 which is <1.52. Therefore, we evaluated Eq (10) for the width of transmission line sections.

The lumped components determined from the Smith chart and the corresponding distributed elements dimensions obtained from mathematical calculations for the 900 MHz converter are listed in Table 1.

**Table 1:** Lumped components and corresponding distributed elements dimensions (mm) for 900 MHz converter.

Lumped Components		Corresponding Distributed Elements
Parameters	Value	Dimensions w×l (${\text{mm}}^{2}$)
Series Inductor *L*_1_	9 nH	2.4 × 30.99
Shunt Capacitor *C*_1_	14.82 pF	2.4 × 52.1
–		2.4 × 5 (Input TL Section)

**Fig 4:**
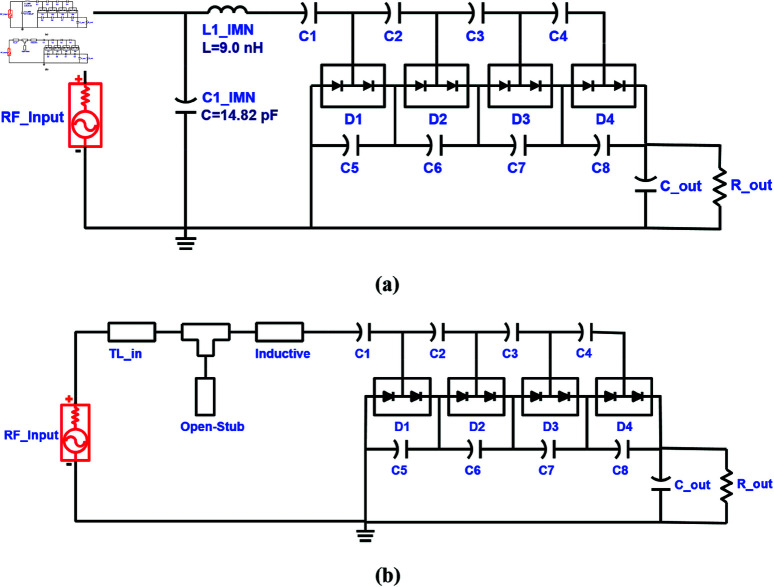
Schematics of RF-to-DC converters working in GSM-900 band utilizing: (a) lumped L-type IMN. (b) Distributed L-type IMN.

The schematics of the single port RF-to-DC converters designed using L-type matching network are shown in Fig 4.

**Fig 5:**
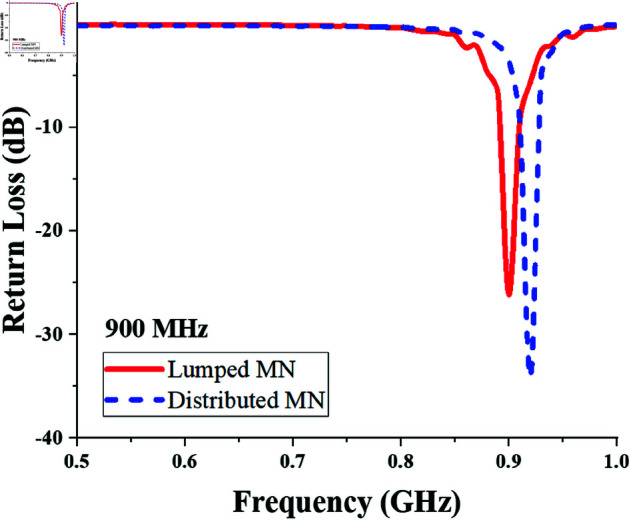
Return loss response of single-port single-band (900 MHz) converter.

The return loss of the 900 MHz single-port single-band converter designed with the lumped and distributed matching networks, is shown in Fig 5. It shows the converter impedance is matched in the required band.

The length of the shunt open stub achieved for 900 MHz converter is approximately λ/4. The open stub offers high impedance at its input, but its impedance becomes low when it resonates. The frequency response of an open stub is a direct function of its impedance, which is directly affected by its length. As the length increases beyond λ/4, stub introduces additional reactive behaviors that make it resonate at more than one frequencies, as more standing waves are produced. This phenomena leads to dual band response of the matching network. Adjustment of the length of the series TL section changes the overall impedance of the network, potentially shifting the resonant frequency and creating a second resonant frequency. The dimensions of the open stub and the series TL section are varied and their behavior is analyzed to achieve dual band matching at 890 and 1850 MHz. The optimized dimensions of the distributed elements of L-matching network of dual band (890 and 1850 MHz) converter, are listed in Table 2.

**Table 2:** Optimized distributed elements dimensions for dual-band (890 and 1850 MHz) converter.

Parameters	Dimensions w×l (${\text{mm}}^{2}$)
Series Inductive TL	2.4 × 19.31
Shunt Open Stub	3.1 × 68.67
Input TL Section	2.4 × 10

**Fig 6:**
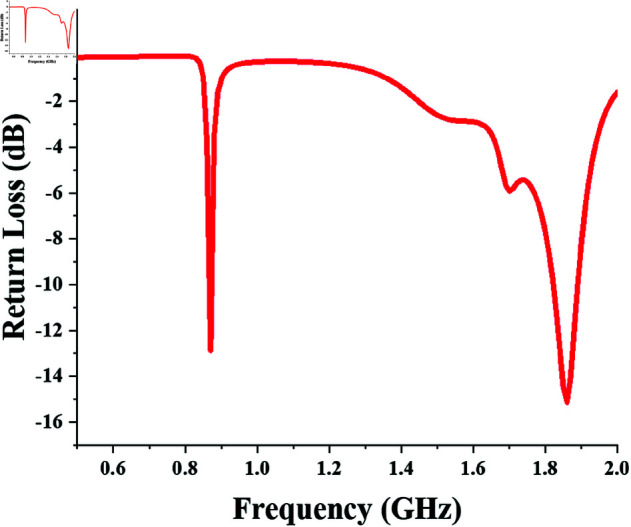
Return loss response of single-port dual-band (890 and 1850 MHz) converter.

Note that the distributed L-type matching network require a TL section at its input (source side) for SMA connector. The return loss response of the resultant single-port dual-band converter is shown in Fig 6 which assures rectification for 890 MHz and 1850 MHz bands.

#### 2.1.2 Single-port triple-band converter design

**Fig 7:**
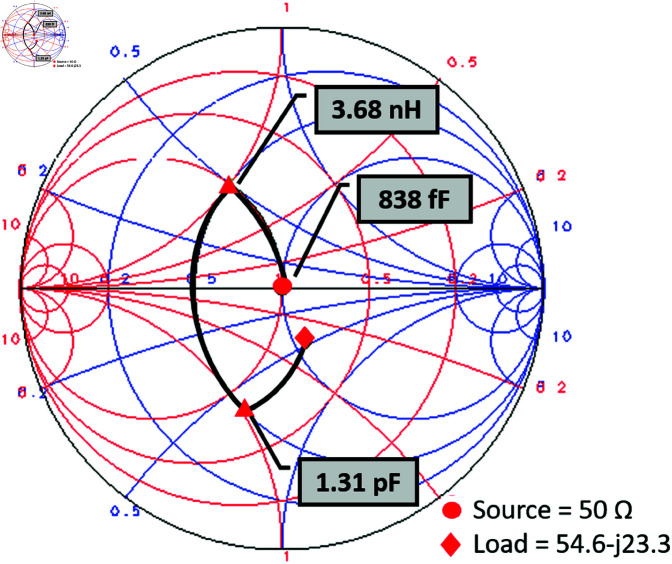
Impedance matching at 2.45 GHz using smith chart.

The proposed single-port triple-band converter design is derived from the modification of a single-port, single-band converter initially operating at 2.45 GHz. To ensure impedance matching between the converter and the input source, a π-matching network, composed of lumped components, was designed using the Keysight ADS Smith Chart utility. The π-matching network is a double stub matching technique consisting of 3 reactive elements, one series inductive while two shunt capacitive (open stubs), elements. The input impedance (Zin) of the converter is noted against 2.45 GHz as 54.65–*j*23.3. The lumped component values of the π-matching network are noted as *L* = 3.678*nH*, *C*_1_ = 1.3139*pF*, and *C*_2_ = 837.9*fF* from the Smith chart as shown in Fig 7.

The lumped element values hence obtained are converted to distributed elements, i.e. the microstrip transmission lines, the same way as done for 900 MHz converter design configuration earlier. The lumped components and corresponding distributed elements dimension obtained for the 2.45 GHz converter are listed in Table 3.

**Table 3:** Lumped components and corresponding distributed elements dimensions (mm) for 2.45 GHz converter.

Lumped components		Corresponding distributed elements
Parameters	Value	Dimensions w×l (${mm}^{2}$)
Series inductor *L*_1_	3.68 nH	2.44 × 12.67
Shunt capacitor *C*_1_	1.13 pF	2.44 × 14.21
Shunt capacitor *C*_2_	838 fF	2.44 × 22.2
–		2.44 × 7 (input TL section)
–		2.44 × 0.1 (output TL section)

**Fig 8:**
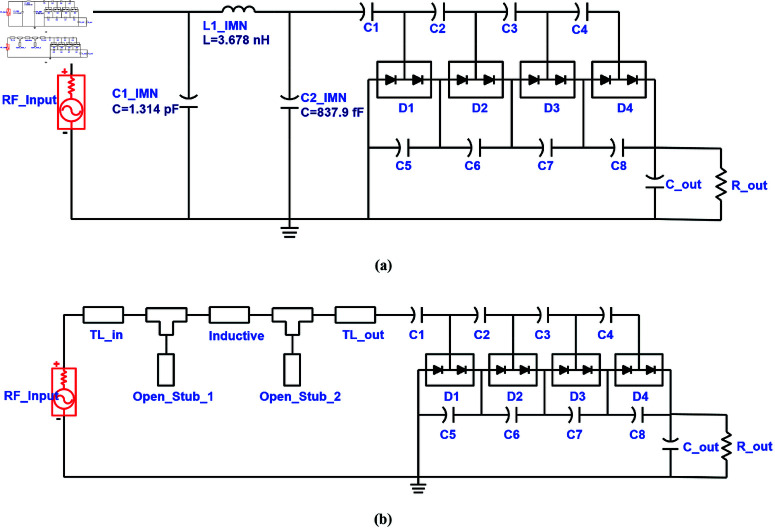
Schematics of RF-to-DC converters working in WiFi-2.45 GHz band utilizing: (a) Lumped π-type IMN. (b) Distributed π-type IMN.

The schematics of the single port RF-to-DC converters designed using π-type matching network are shown in Fig 8.

**Fig 9:**
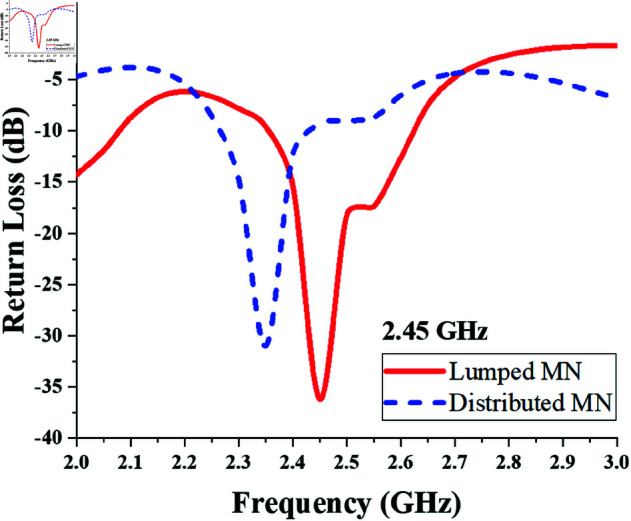
Return loss response of single-port single-band (2.45 GHz) Converter.

The resulting return loss of the 2.45 GHz converter designed with lumped and distributed matching networks is shown in Fig 9. It shows the achievement of converter impedance matching in the required band.

Further, the dimensions of the distributed matching elements are optimized to obtain the triple band frequency response. The length of series inductive TL section is slightly increased to introduce additional phase shift and impedance transformation. The shunt open stubs are modified to a shorter lengths to introduce resonance at two additional frequencies (1.775 and 2.25 GHz), lower than 2.45 GHz. Therefore the careful optimization and analysis of the distributed elements dimensions resulted in the achievement of triple band matching response form π-matching network without introducing additional components. The optimized dimensions of the distributed elements of π-matching network of triple band (1775 MHz, 2.25 GHz, and 2.45 GHz) converter, are listed in Table 4.

**Table 4:** Optimized distributed elements dimensions for triple band (1775 MHz, 2.25 GHz, and 2.45 GHz) converter.

Parameters	Dimensions w×l (${mm}^{2}$)
Series inductive TL	2.4 × 14.67
Shunt open stub 1	2.4 × 5.9
Shunt open stub 2	2.35 × 10.4
Input TL section	2.36 × 5
Output TL section	2.37 × 19.95

**Fig 10:**
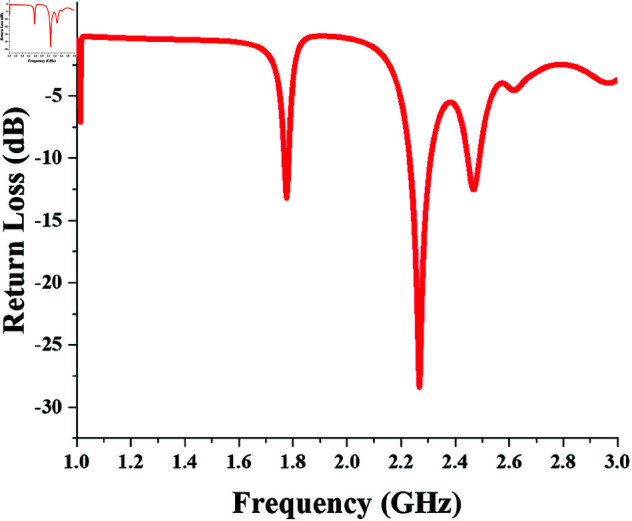
Return loss response of single-port triple-band (1775 MHz, 2.25 GHz, and 2.45 GHz) converter.

Note that the distributed π-matching network requires a TL section at input (source side) for SMA connector and also at its output (load side) to be connected with rectification block elements. The triple band converter return loss is shown in Fig 10. It shows that the triple-band converter is capable to provide rectification for the 1775 MHz, 2.25 GHz, and 2.45 GHz bands.

The subsequent milestone involves the derivation of a dual-port hexa-band converter from two single-port converters: a dual-band converter and a triple-band converter. The intricate design considerations for the dual-port converter are elaborated upon in the subsequent subsection.

### 2.2 Dual-port hexa-band converter design

**Fig 11:**
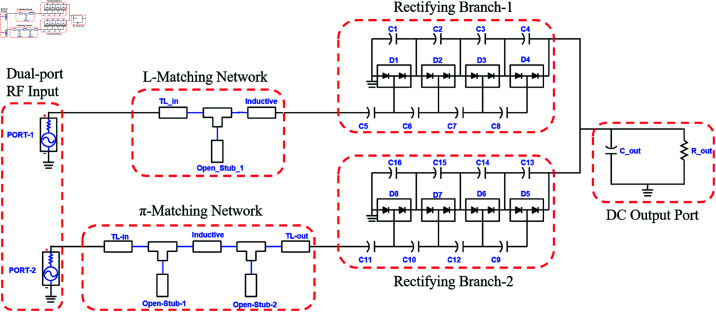
Hexa band converter schematic.

The single-port dual-band and the triple-band converters are integrated to obtain a hexa-band converter comprising two input ports and a single output port. The port-1 is matched with the rectification block, incorporating an L-matching network, while port-2 is matched using a π-matching network. The schematic of the hexa-band converter is shown in Fig 11.

**Fig 12:**
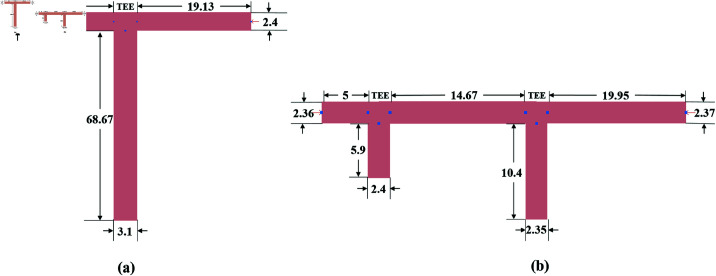
Proposed impedance matching networks. (a) L-type matching network. (b) π-Type matching network.

Each of the IMN branches consists of microstrip transmission lines and open stubs, working on the required tuned frequencies. Transmission lines and stubs are interconnected by the microstrip TEEs. The layout of the IMNs and their dimensions are shown in Fig 12. All the dimensions are mentioned in millimeters (mm).

**Fig 13:**
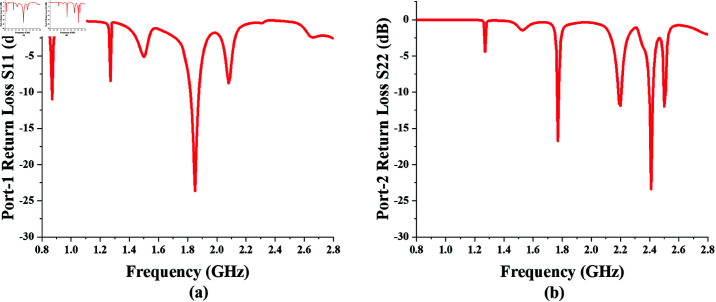
Return Loss for Input Ports of the Proposed converter. ( **a**) Return Loss for Port-1. ( **b**) Return Loss for Port-2.

The return loss for each input port is shown in Fig 13. It can be seen that the rectifying branch-1, that is energized via port-1, provides rectification for the 870 and 1850 MHz bands and gives a dual-band response. However, rectifying branch-2, which is energized via port-2, provides a quad-band response at frequencies 1.770, 2.2, 2.4, and 2.5 GHz. The S-parameter at these frequencies is fairly below the –10 dB level.

**Fig 14:**
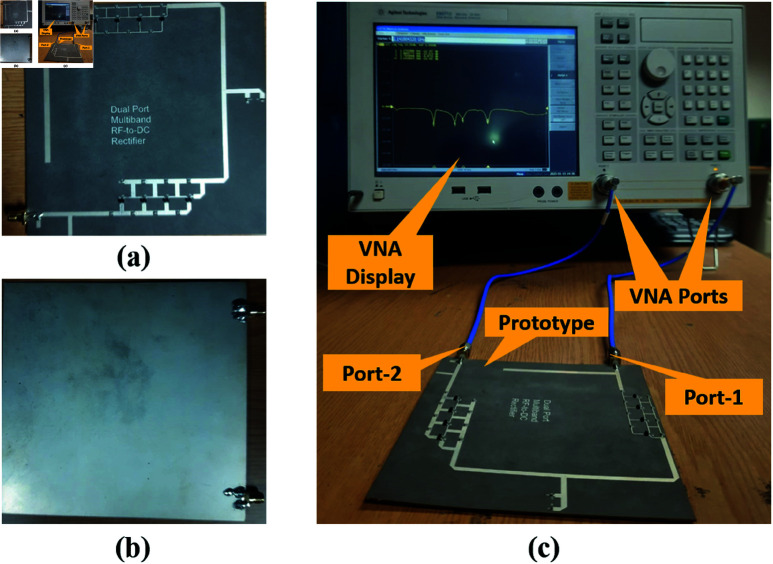
Prototype performance validation. ( **a**) Top view. ( **b**) Bottom view. ( **c**) Experimental setup.

The prototype of the converter is fabricated using an 18 kΩ output resistance and its return loss response is noticed on the virtual network analyzer (VNA). Fig 14 shows the top and bottom view of the prototype and the experimental setup established for the converter performance validation.

This research primarily involved theoretical circuit design, simulation, analysis and in-Lab testing and verification. As no field work, animal experimentation, or human subject research was conducted, therefore no specific permits were required. The research utilized commercially available software tools and readily accessible information on electromagnetic radiation and circuit components.

## 3 Results and discussion

1The proposed converters have been simulated for different values of input power (*P*_*in*_) and output load resistance (*R*_*out*_). For each simulation, the corresponding values of the output DC voltage $V_{out}$ are noticed. The output voltage is observed to increase with increasing *R*_*out*_ and *P*_*in*_. The efficiency of the converter is given as Eq (13):

$$\eta =\frac{P_{out}}{P_{in}},$$ (13)

where η is the converter efficiency, *P*_*out*_ is the output DC power and the *P*_*in*_ is the RF input power. The output DC power can be calculated as Eq (14):

$$P_{out}=\frac{V_{out}^{2}}{R_{out}},$$ (14)

Therefore, the efficiency η can be mentioned by the Eq (15):

$$\eta =\frac{V_{out}^{2}}{P_{in}\times R_{out}},$$ (15)

### 3.1 Single port converters analysis

**Fig 15:**
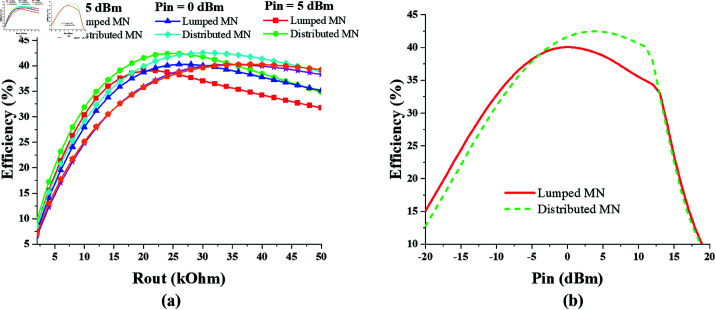
Single-port single-band GSM-900 converter efficiency performance against varying (a) *R*_*out*_. (b) *P*_*in*_.

To determine the optimum value of the output resistance *R*_*out*_, the efficiencies of the converters are recorded for different values of the *R*_*out*_ keeping *P*_*in*_ constant. The efficiency versus *R*_*out*_ graphs of the single-port single-band GSM-900 converter are shown in Fig 15a, using the lumped MN as well as the distributed MN. The optimum range of *R*_*out*_ is 22−26kΩ, and the peak efficiency of 40.3% and 42.5% is achieved with lumped MN and distributed elements MN respectively. The efficiency response of the converter against varying levels of input power Pin is shown in Fig 15b. It shows that converter gives the peak efficiency when input power is 5 dBm and output resistance is 24kΩ. It is also concluded that distributed matching network gives a slightly higher efficiency than lumped element matching.

**Fig 16:**
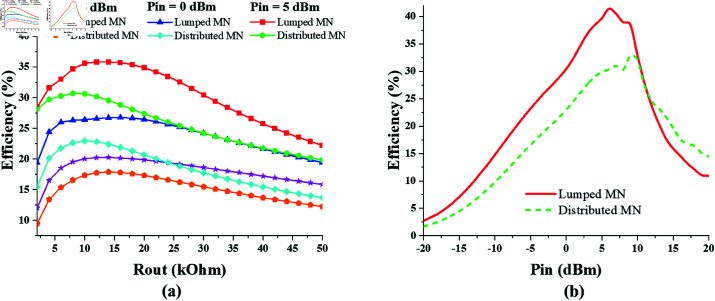
Single-port single-band 2.45 GHz converter efficiency performance against varying (a) *R*_*out*_. (b) *P*_*in*_.

Similarly, the optimum output resistance of single-port single-band 2.45 GHz converter is extracted from the analysis of its efficiency versus *R*_*out*_ graphs shown in Fig 16a. The optimum *R*_*out*_ range is 10−14kΩ , where the converter provides peak efficiency levels. Hence considering 12kΩ as optimum *R*_*out*_, the efficiency response of the converter against varying *P*_*in*_ levels is shown in Fig 16b. The converter peak efficiency is 41.4% when lumped components are used for matching, and 32.8% when the distributed elements are employed for matching.

**Fig 17:**
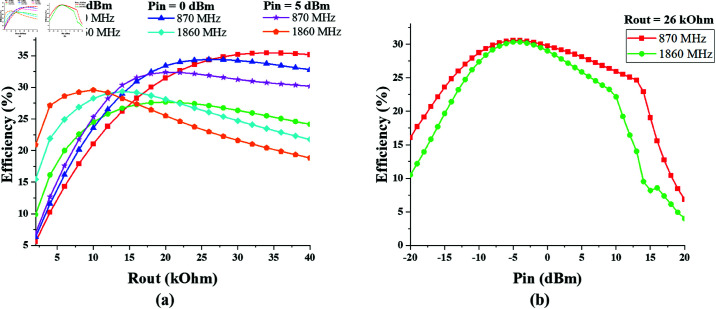
Single-port dual-band converter efficiency performance against varying, (a) *R*_*out*_. (b) *P*_*in*_.

The optimum output resistance for the single-port dual-band (890 MHz, and 1850 MHz) converter is extracted from its efficiency versus *R*_*out*_ graphs shown in Fig 17a. It can be seen that maximum efficiency level of about 30% is obtained when the Rout is kept 26 kΩ at –5 dbm of input power. The efficiency versus *P*_*in*_ curve is also shown in Fig 17b when Rout is kept 26 kΩ.

**Fig 18:**
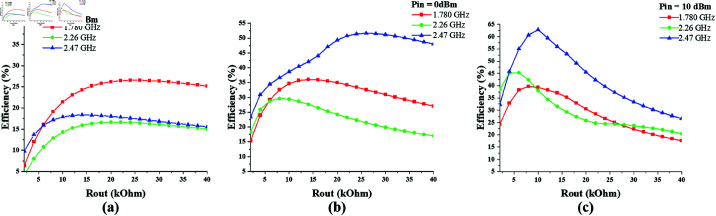
Single-port triple-band converter efficiency vs *R*_*out*_ curve at constant input power. (a) *P*_*in*_ = –10 dBm. (b) *P*_*in*_ = 0 dBm. (c) *P*_*in*_ = 10 dBm.

Likewise, to determine the optimum value of the output resistance of the single-port triple-band (1775 MHz, 2.25 GHz, and 2.45 GHz) converter, its efficiency response is noted against varying values of *R*_*out*_, as shown in Fig 18. It shows that the converter response is appreciable in the range of 10 – 15 kΩ Rout. The peak efficiency of 64% is achieved at 2.47 GHz when *R*_*out*_ is 12 kΩ and *P*_*in*_ is 10 dBm.

**Fig 19:**
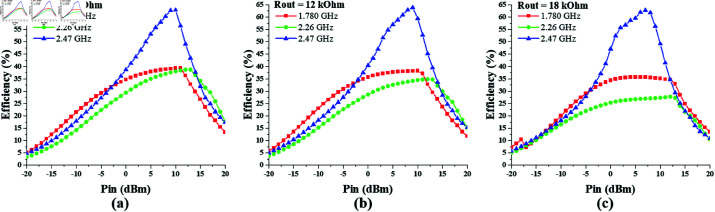
Single-port triple-band converter efficiency vs *P*_*in*_ curve at different *R*_*out*_ values. (a) *R*_*out*_ = 10  kΩ. (b) *R*_*out*_ = 12 kΩ. (c) *R*_*out*_ = 18 kΩ.

Fig 19 shows the efficiency response of single-port triple-band converter against varying input power, when output resistance is 10, 12 and 18 kΩ respectively. The maximum efficiency levels are recorded when the input power is in the range of 5–10 dBm.

### 3.2 Dual-port hexa-band converter analysis

**Fig 20:**
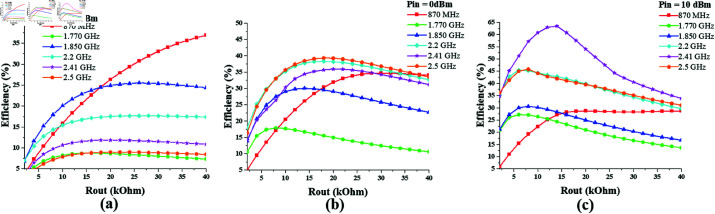
hexa band converter efficiency vs *R*_*out*_ curve at constant input power. ( **a**) *P*_*in*_ = –10 dBm. ( **b**) *P*_*in*_ = 0 dBm. ( **c**) *P*_*in*_ = 10 dBm.

The optimum value of the output resistance *R*_*out*_ for the dual-port hexa-band converter design is determined by the analysis of its efficiency response against varying *R*_*out*_, while keeping the input power constant, as shown in Fig 20. It shows that the peak efficiencies are obtained when the output resistance value is in the range 10–20 kΩ. moreover the maximum PCE is achieved when the input power is 10 dBm (10 mW).

**Fig 21:**
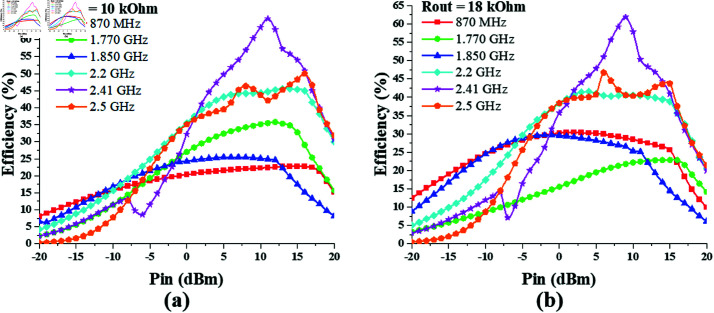
Hexa band converter efficiency vs *P*_*in*_ curve at different rout values. (a) *R*_*out*_ = 10 kΩ. (b) *R*_*out*_ = 18 kΩ.

The efficiency vs *P*_*in*_ curve for the proposed dual-port hexa-band converter is displayed in Fig 21. It shows that the efficiency of the converter increases with the increase in input power level and attains a peak at certain value of *P*_*in*_, after which is starts decreasing. Appreciable efficiencies are noted against 10 kΩ and 18 kΩ of output resistance.

From the analysis of the dual-port hexa-band converter responses against varying *P*_*in*_ levels and varying values of *R*_*out*_, it can be seen that the converter attains peak efficiency levels of 66% and 62% for 10 kΩ and 18 kΩ output resistance, respectively. The converter peak efficiency responses are summarized in Table 5.

**Table 5:** Summarized dual-port hexa-band converter peak efficiency performance.

Rout	10 (kΩ)			18 (kΩ)		
Frequency	η (%)	$V_{out}$ (V)	$P_{in}$ (dBm)	η (%)	$V_{out}$ (V)	$P_{in}$ (dBm)
870 MHz	22.7	4.49	15	30.2	2.33	0
1770 MHz	35.5	6.69	11	23	11.41	15
1850 MHz	25.5	3.5	6	29.7	2.1	–1
2.2 GHz	45.5	10.7	14	41.5	4.33	4
2.41 GHz	66	9.13	11	62	9.4	9
2.5 GHz	46.4	5.41	8	46.7	5.8	6

The proposed design is compared with some recent RF-to-DC converter designs presented in the literature to highlight significance of the proposed converter. The comparison is summarized in Table 6. It can be seen that the proposed converter has achieved hexa-band rectification with comparable PCE levels. The proposed design’s performance outshines in terms of its multiband PCE by achieving simultaneous impedance matching in diverse frequency bands of the two port converter.

**Table 6:** Comparison of proposed converter with some other designs in literature.

Ref.	Band (GHz)	η (% )	$P_{in}$ (dBm)	$R_{out}$ (kΩ )	$V_{out}$ (V)
[16]	0.900	29.85	20		17.58
[35]	2.5	33.5	5		1.89
[20]	1.8	41	0	30	
	2.45	20			
[8]	0.900	33.7	–10	3.8	0.02–3.8
	1.8	21.8			
	2.45	20			
[36]	0.89	60			0.19–12.43
	1.83	48			
	2.19	35			
	2.45	25			
[27]	0.55	–		10–75	
	0.75	–			
	0.9	67			
	1.85	–			
	2.15	–			
	2.45	–			
[28]	1.84	44.4	–10		
	2.04	43.9			
	2.36	45.4			
	2.54	43.4			
	3.3	36.1			
	4.76	32.4			
	5.8	28.3			
Proposed work with 10 kΩ *R*_*out*_	0.87	22	10	10	5.3–9.13
	1.770	35			
	1.850	25			
	2.2	44.5			
	2.41	63.6			
	2.5	44			
Proposed work with 18 kΩ *R*_*out*_	0.87	29	9	18	2.1–11.4
	1.770	22			
	1.850	26.5			
	2.2	40.6			
	2.41	62			
	2.5	40.6			

## 4 Conclusion

This article explores a Dual-Port Multiband RF-to-DC converter designed to meet dynamic energy needs of IoT devices. The innovative design on a 0.78 mm thick Rogers-5880 substrate demonstrates adaptability for diverse IoT environments. By harnessing RF energy from 870 MHz, 1770 MHz, 1850 MHz, 2.2 GHz, 2.4 GHz and 2.5 GHz bands simultaneously, it significantly boosts energy acquisition. The proposed circuitry and impedance matching techniques are used to optimize conversion efficiency and output voltage across all the desired RF frequencies, simultaneously. Systematic analysis of parameters like output voltage and conversion efficiency provides valuable performance insights. The impressive efficiency figures are observed across different frequency bands (0.87–2.5 GHz) for 10 kΩ as well as 18 kΩ output resistance. The proposed design exhibits peak efficiency of 66% and 62% at 10 kΩ and 18 kΩ, respectively. Comparative analysis with recent converter designs highlights its multiband capability, impressive efficiency, innovative impedance matching techniques, competitiveness and innovation for energy-efficient IoT solutions. This work holds great promise for practical implementation in various IoT environments, ultimately facilitating the growth of sustainable and autonomous IoT ecosystems.
